# Redox dependent metabolic shift in *Clostridium autoethanogenum* by extracellular electron supply

**DOI:** 10.1186/s13068-016-0663-2

**Published:** 2016-11-16

**Authors:** Frauke Kracke, Bernardino Virdis, Paul V. Bernhardt, Korneel Rabaey, Jens O. Krömer

**Affiliations:** 1 Centre for Microbial Electrochemical Systems, The University of Queensland, Brisbane, QLD 4072 Australia; 2 Advanced Water Management Centre, The University of Queensland, Brisbane, QLD 4072 Australia; 3 School of Chemistry and Molecular Biosciences, University of Queensland, Brisbane, QLD 4072 Australia; 4 Laboratory of Microbial Ecology and Technology, Faculty of Bioscience Engineering, Universiteit Ghent, Ghent, Belgium

**Keywords:** Microbial electrosynthesis, Bio production, Gas fermentation, Wood-Ljungdahl pathway, Extracellular electron transport, Redox mediator, Acetogen, Rnf complex

## Abstract

**Background:**

Microbial electrosynthesis is a novel approach that aims at shifting the cellular metabolism towards electron-dense target products by extracellular electron supply. Many organisms including several acetogenic bacteria have been shown to be able to consume electrical current. However, suitable hosts for relevant industrial processes are yet to be discovered, and major knowledge gaps about the underlying fundamental processes still remain.

**Results:**

In this paper, we present the first report of electron uptake by the Gram-positive, ethanol-producing acetogen, *Clostridium* *autoethanogenum*. Under heterotrophic conditions, extracellular electron supply induced a significant metabolic shift away from acetate. In electrically enhanced fermentations on fructose, acetate production was cut by more than half, while production of lactate and 2,3-butanediol increased by 35-fold and threefold, respectively. The use of mediators with different redox potential revealed a direct dependency of the metabolic effect on the redox potential at which electrons are supplied. Only electrons delivered at a redox potential low enough to reduce ferredoxin caused the reported effect.

**Conclusions:**

Production in acetogenic organisms is usually challenged by cellular energy limitations if the target product does not lead to a net energy gain as in the case of acetate. The presented results demonstrate a significant shift of carbon fluxes away from acetate towards the products, lactate and 2,3-butanediol, induced by small electricity input (~0.09 mol of electrons per mol of substrate). This presents a simple and attractive method to optimize acetogenic fermentations for production of chemicals and fuels using electrochemical techniques. The relationship between metabolic shift and redox potential of electron feed gives an indication of possible electron-transfer mechanisms and helps to prioritize further research efforts.

**Electronic supplementary material:**

The online version of this article (doi:10.1186/s13068-016-0663-2) contains supplementary material, which is available to authorized users.

## Background

Microbial electrosynthesis (MES) is a bio-electrochemical technology describing the microbial conversion of CO_2_ or cheap carbon sources and electrical energy into valuable chemicals and fuels. A great driving force of MES technologies is its potential as carbon-capture process to significantly contribute to lower CO_2_ emissions and therefore have a positive effect on climate change [1]. For a long time, the use of CO_2_ as a raw material was deemed unfeasible as the gas is difficult to capture from the atmosphere [2]. In recent years, there has been a dramatic shift of this opinion, however, as various industries today provide gaseous waste-streams rich in CO_2_, thus making the compound easily available and facilitating CO_2_-utilizing technologies to be applied. For instance, the biotech company LanzaTech constantly advances available tools for metabolic and genetic engineering as well as fundamental understanding of the acetogenic metabolism [3, 4] and specializes in gas-fermentation processes in which the autotrophic acetogen *Clostridium autoethanogenum* converts the waste gas of steel mill plants into acetate and ethanol [5]. The electrons for CO_2_ reduction in this process are provided by carbon monoxide and hydrogen that are also present in the waste gas. MES technologies could dramatically increase the usable spectrum of CO_2_-rich gases by providing additional electrons via an electrochemical route and therefore optimizing the electron-to-CO_2_ ratios. The economic viability of such a process would be determined mostly by the cost of the electricity used and the product revenue [6]. A process that aims at using CO_2_ as sole carbon source (no CO) and delivering all electrons via an electrochemical route requires high charge densities, which remains a challenge to achieve in cathodic processes to date [7]. However, bio-electrochemical production technologies are not limited to the use of CO_2_, and many approaches focus on the use of organic molecules from other industrial waste streams or sustainable sources as substrates for MES, which is often titled electro-fermentation [8, 9].

A major challenge for the research community is the identification of suitable hosts for MES and understanding the fundamental processes of electron transfer to optimize electron-uptake rates. Acetogenic organisms are regarded attractive hosts for microbial electrosynthesis since they can use a variety of compounds as sources of energy and carbon. Reduction of carbon dioxide is possible via the Wood-Ljungdahl pathway (WLP), which is the most efficient microbial pathway for CO_2_-fixation [10, 11]. The low energy efficiency of WLP still has beneficial effects, as it results in high electron recovery in products and low biomass production [11]. The main product is usually acetate, but the central intermediate acetyl-CoA presents an excellent building block for various commodities [12, 13]. Tools for genetic manipulation of acetogenic organisms are rapidly evolving, offering a fast-growing set of techniques for pathway engineering [14]. Electrochemical activity has been reported for several acetogens that perform MES by direct electron uptake from the electrode [15] or mediated either via electrochemically produced hydrogen [16] or via added redox compounds such as methyl viologen [17]. These redox compounds include cytochrome-containing organisms such as *Moorella thermoacetica* as well as *Clostridium ljungdahlii* which contains no cytochromes.

The mechanisms of extracellular electron transport in acetogens remain to be uncovered. However, a detailed discussion of metabolic electron transfer and possible interactions with an electrode are discussed in a preceding work from our group [18]. By analysing the electron feed from a cathode to the cellular metabolism theoretically, we identified the entry point of electrons into the microbial metabolism as a step of major importance for MES processes [18, 19]. It was shown that depending on the metabolic reaction that is directly related to extracellular electron supply, different effects on cellular redox and energy yields might be achieved. Therefore, this study follows an approach of electron supply via mediators of different midpoint potentials with the aim to target different points of metabolic electron transfer within the cells. The redox mediators of choice include compounds with midpoint redox potentials lower than that of typical mediators used thus far in bio-electrochemical technologies (e.g. neutral red or methyl viologen), which thus could offer a thermodynamic advantage for the microbes.

Amongst the first acetogens to be applied in a bio-electrochemical process for production was *C.* *ljungdahlii*, and quickly it was titled a “chassis organism” for electrified reduction of CO_2_ as it is one of the few acetogens with a fully sequenced genome as well as available tools for metabolic engineering [10, 13]. Its close relative, *C.* *autoethanogenum,* has not been tested in a bio-electrochemical environment previously, but is considered an industrial model gas-fermenting acetogen offering sustainable bio-production of commodities and low-carbon fuels by means of C_1_ feedstocks from waste gases such as syngas [3]. The attractive feature about *C.* *autoethanogenum* is its natural increased formation of ethanol compared with other acetogens, which presents a higher value product than acetate [20].

In a first step towards microbial electrosynthesis from CO_2_, we investigate the optimal energy levels for electron delivery to the metabolism. This is achieved by characterizing the electrochemical activity of *C.* *autoethanogenum* and the effect of extracellular electron supply at different potentials on the product spectrum on fructose.

## Results

Hydrogen is a favourable electron donor in natural environments for *C.* *autoethanogenum* [20]. Under the hypothesis that in order to be able to feed to the electron transport chain of *C.* *autoethanogenum,* soluble redox-mediating molecules would need to enter the cells at an energy level similar to that ofas H_2_, we have selected three redox-active molecules with midpoint potential around that of the couple H^+^/H_2_. Under the standard conditions (25 °C, pH = 7), the redox potential for its electrochemical formation is −414 mV [vs the standard hydrogen electrode, SHE (note: all potentials herein are reported with respect to this reference electrode)]. However, the low-hydrogen partial pressure in acetogenic environments can shift this potential more to values around −350 mV [21].

**Fig. 1:**
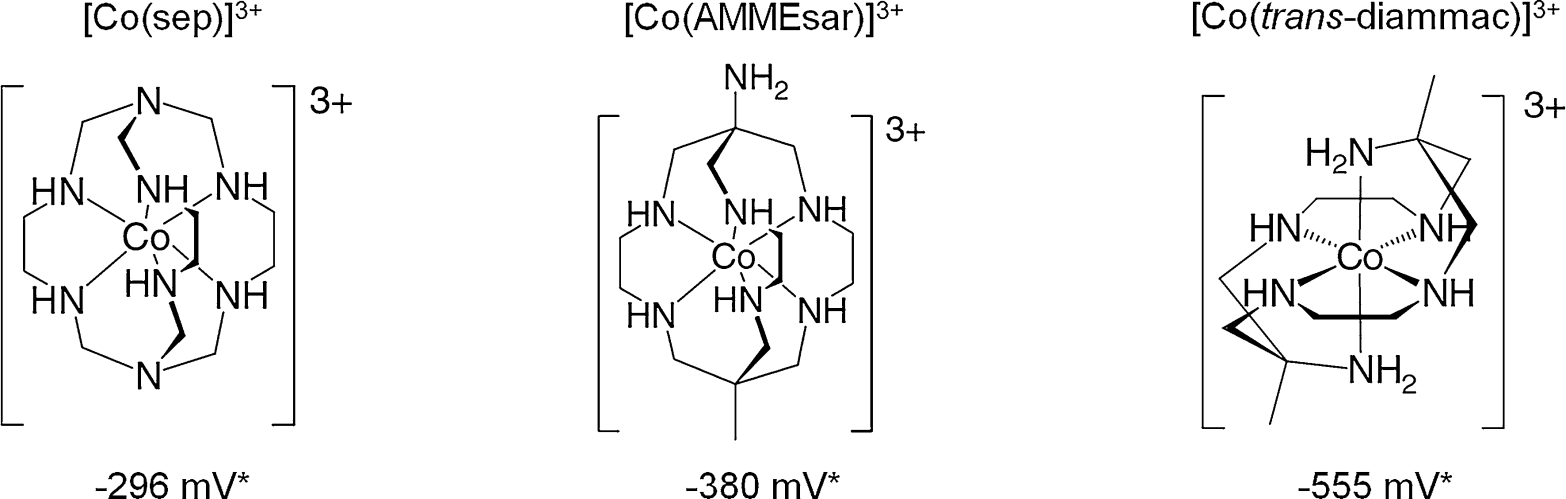
Chemical structures of the three macrocyclic cobalt hexamines used as redox mediators in this study. *E^0’^ vs SHE at 25 °C and pH 7 [22]

**Fig. 2:**
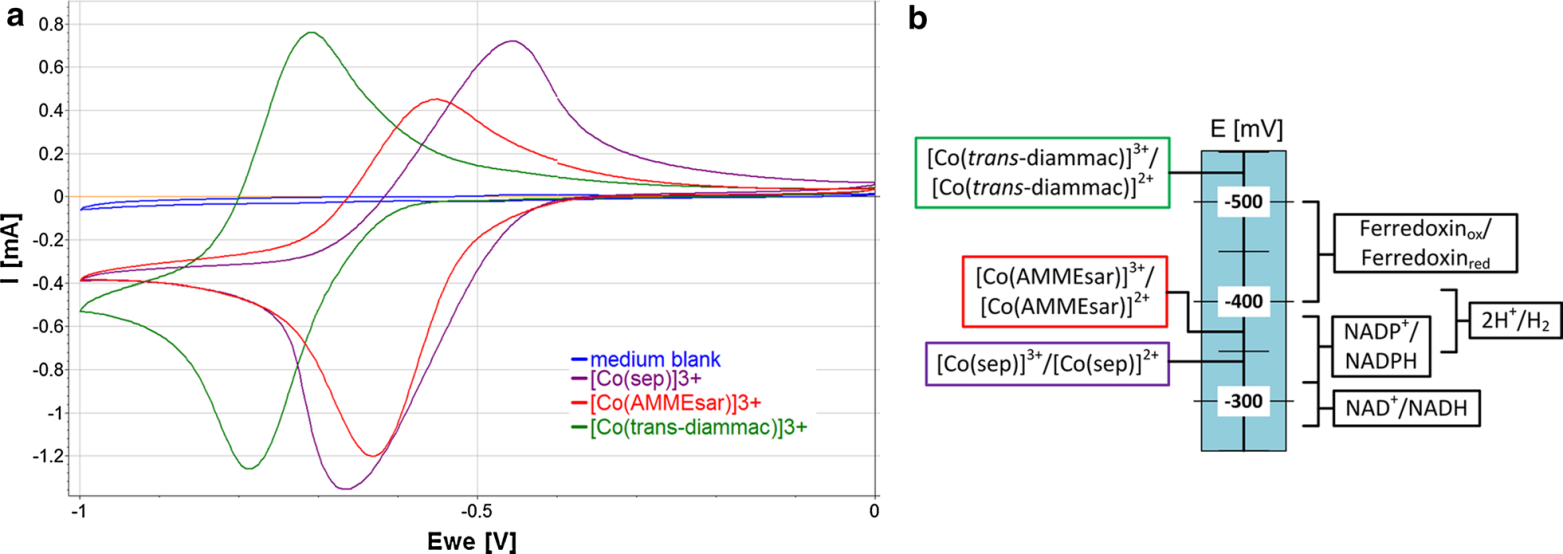
Redox potentials of the three mediators [Co(sep)]^3+^, [Co(AMMEsar)]^3+^ and [Co(*trans*-diammac)]^3+^. **a** Cyclic voltammograms recorded in the bio-electrochemical system at 1 mV/s for 5 cycles, E_WE_ vs Ag/AgCl. **b** Schematic image of mediator redox potentials and redox windows for biological reduction of ferredoxin, NAD(P)^+^ and H^+^

The three redox-active soluble molecules were cobalt-based complexes [Co(sep)]^3+^ (Co^III/II^ −296 mV), [Co(AMMEsar)]^3+^ (Co^III/II^ −380 mV) and [Co(*trans*-diammac)]^3+^ (Co^III/II^ −555 mV) (Fig. 1) previously used in redox reactions with proteins [22, 23]. These compounds are very stable (in both their Co^III^ and Co^II^ oxidation state) and comprise bio-compatible molecules that offer the advantage of having extremely similar chemical and physical properties. Therefore, their actual Co^III/II^ redox potentials are the only significant variable. These were determined by cyclic voltammetry under the given conditions in the bio-electrochemical system (BES) (carbon cloth working electrode, fermentation medium, pH = 5.9, 37 °C) to be [Co(sep)]^3+^ −340 mV, [Co(AMMEsar)]^3+^ −370.5 mV and [Co(*trans*-diammac)]^3+^ −526.5 mV (Fig. 2a). The values are in good agreement with given mid-point potentials at pH = 6 in the literature with deviations of −30, +1.5 and −11.5 mV, respectively [22]. These small variations are most likely due to ion pairing with buffer anions in the medium. Figure 2b illustrates the midpoint potentials of the mediators in the context of the redox potentials for the ferredoxin_ox/red_, NAD(P)H/NAD(P)^+^ and H_2_/H^+^ couples, which act as important redox co-factors in the metabolism of *C.* *autoethanogenum*.

The mediators were reduced electrochemically in the BES prior to inoculation. In all fermentations, a constant potential of −603 mV was applied, which was sufficiently negative to reduce all of the mediators to their Co^II^ state while avoiding hydrogen evolution on the carbon cloth working electrode (as suggested by the absence of a catalytic wave for hydrogen evolution at that potential, determined by CV, see Fig. 2a). The system was inoculated with *C.* *autoethanogenum* after a stable baseline current was achieved.

**Fig. 3:**
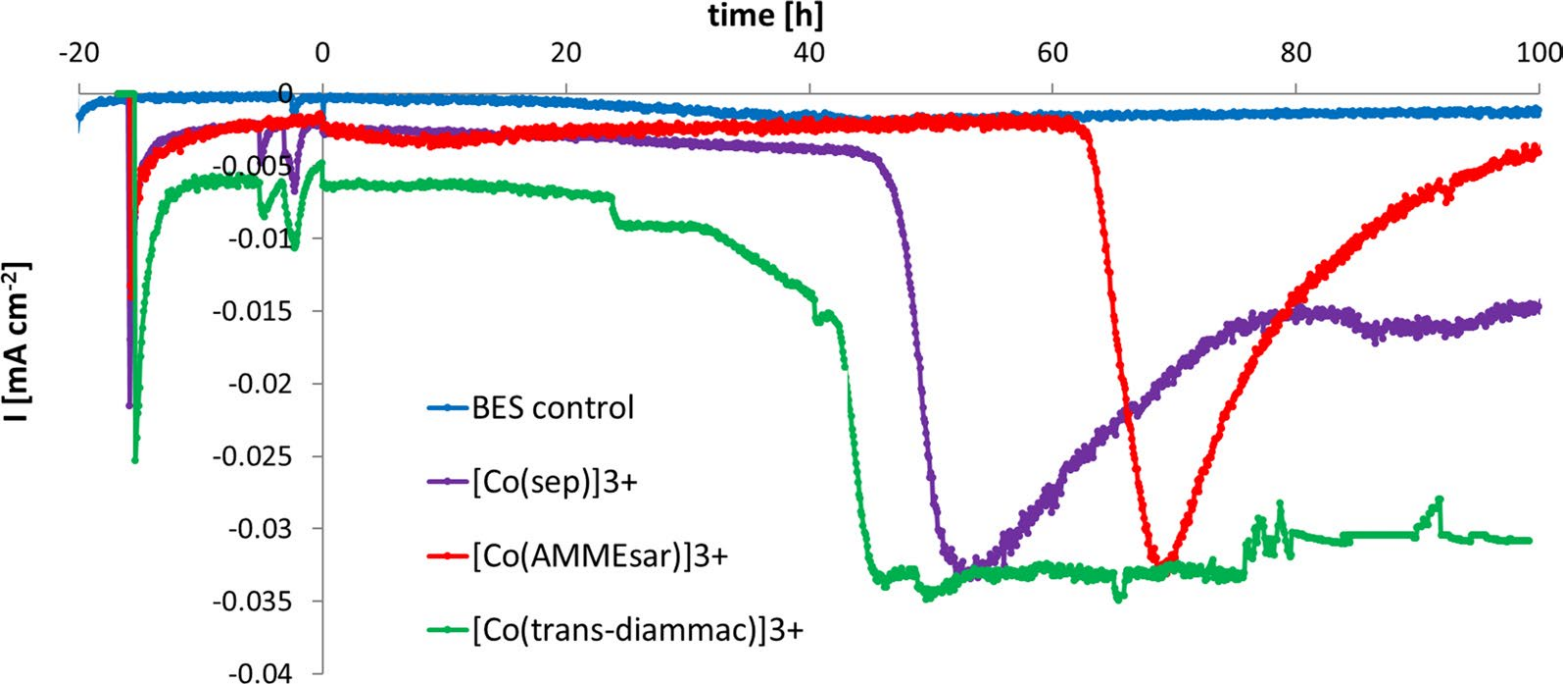
Current profiles of *Clostridium autoethanogenum* fermentations on fructose in the bio-electrochemical system in the absence (control) and in the presence of the three mediators [Co(sep)]^3+^, [Co(AMMEsar)]^3+^ and [Co(*trans*-diammac)]^3+^. Concentration of the mediators was 1 mmol/L and a constant potential of −0.603 V (vs SHE) was applied on the working electrode. Negative time points correspond to the phase of abiotic mediator reduction prior to inoculation

**Fig. 4:**
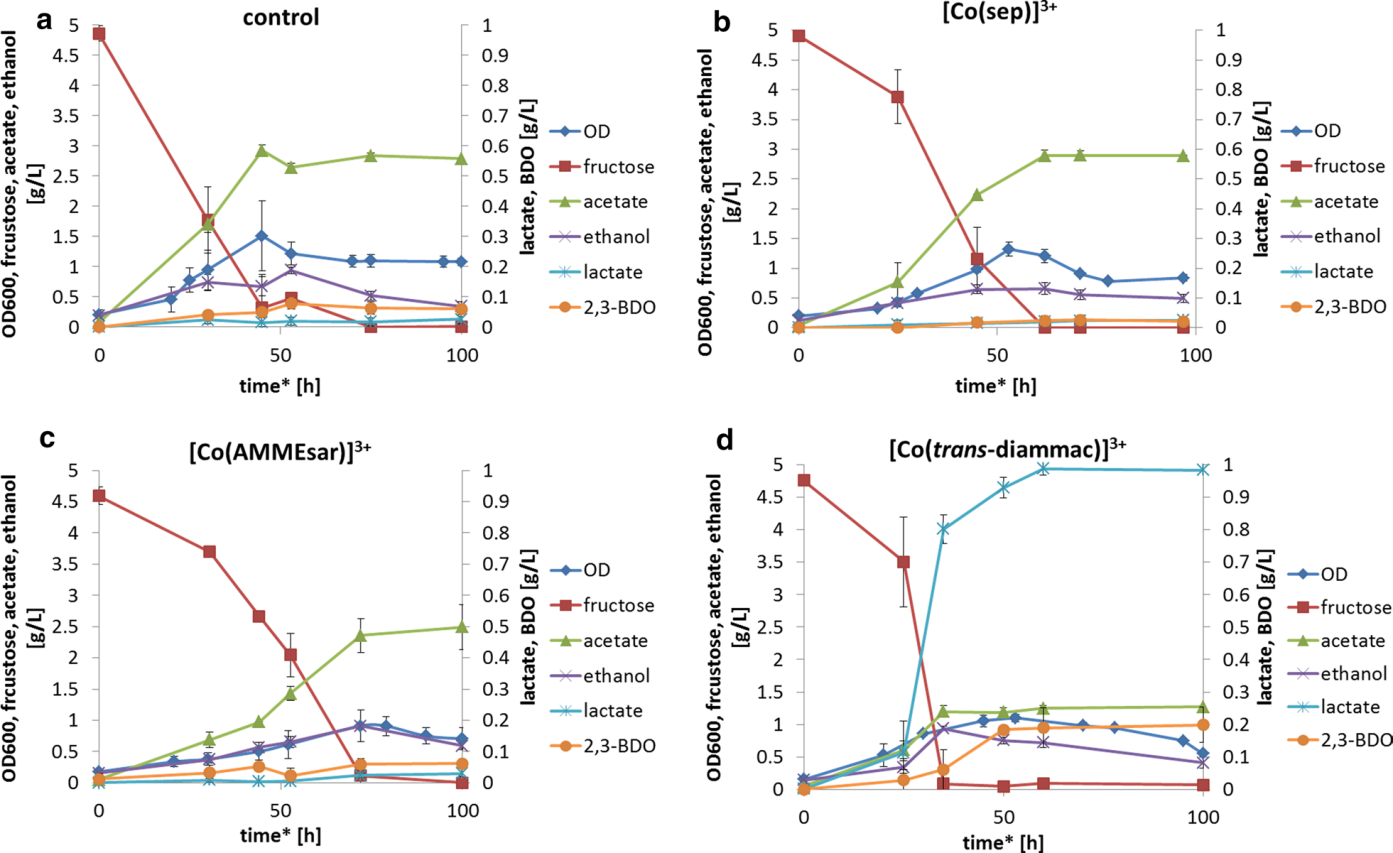
Metabolic profiles of heterotrophic anaerobic fermentations of *C.* *autoethanogenum* in the absence (**a** control) and in the presence of the three mediators **b** [Co(sep)]^3+^, **c** [Co(AMMEsar)]^3+^ and **d** [Co(*trans*-diammac)]^3+^. Concentration of the mediators was 1 mmol/L, and a constant potential of −0.603 V (vs SHE) was applied throughout the experiment. Control fermentations were performed in the same reactor set up with electrodes at open circuit. Shown data are average values of triplicate fermentations with error bars displaying the corresponding standard deviations. *Fermentation times are adjusted to time zero = start of fructose consumption as varying lag-phases in the presence of the mediators were observed.* 2,3-BDO* 2,3-butanediol,* OD* optical density

In heterotrophic, anaerobic fermentations (C-source: fructose, 5 g/L) of *C.* *autoethanogenum,* current consumption in the presence of all three mediators was observed. Current profiles in the presence and the absence of the mediators are shown in Fig. 3. Importantly, no significant current was detected in controls without mediator or without cells. Further, a metabolic effect due to the presence of the compounds was ruled out by fermentations with the mediators at open circuit, which had no effect on production or growth (data not shown). After inoculation in the BES, varying lag phases were observed in the presence of the mediators (maximum 20 h for [Co(*trans*-diammac)]^3+^), and therefore, time zero was defined as the starting point of fructose consumption. Figure 4 shows the metabolic profiles of mediated fermentations in the BES. Applying potential to the cathode resulted in, in all cases, slightly lower maximum optical densities (OD_max_): 1.51 ± 0.18 in control fermentations and 1.11 ± 0.03, 1.3 ± 0.12, 1.22 ± 0.22 and 1.09 ± 0.14 in the BES without mediator and in the presence of [Co(sep)]^3+^, [Co(AMMEsar)]^3+^ and [Co(*trans*-diammac)]^3+^, respectively. As seen from Fig. 4, very little change in the final end-products of fructose fermentation was made by [Co(sep)]^3+^ (Fig. 4b) and [Co(AMMEsar)]^3+^ (Fig. 4c) compared with the control (Fig. 4a). Over a duration of 100 h, control fermentations produced 2.84 ± 0.29 g/L acetate as main product. Fermentations with [Co(sep)]^3+^ showed similar behaviour with final acetate concentrations of 2.90 ± 0.10 g/L. [Co(AMMEsar)]^3+^ seemed to slightly slow down substrate uptake and growth rate (*µ*_max,control_ = 0.053 ± 0.006/h^−1^, *µ*_max,[Co(AMMEsar)]_^3+^ = 0.033 ± 0.006/h^−1^). However, final concentrations of metabolites remained at similar levels with final acetate concentrations of 2.49 ± 0.36 g/L. Only the low-potential mediator significantly changed the metabolite spectrum. In fact, in the presence of 1 mmol/L [Co(*trans*-diammac)]^3+^, acetate production decreased sharply (from 2.84 ± 0.29 to 1.27 ± 0.02 g/L). Simultaneously, lactate and 2,3-butanediol productions were enhanced from 0.03 ± 0.01 to 0.98 ± 0.02 g/L and from 0.06 ± 0.01 to 0.2 ± 0.05 g/L, respectively (see Fig. 4d).

Table 1 summarizes in detail the parameters of metabolite concentrations, yields, growth and electron consumption in fermentations with and without the three mediators. The amount of electrons consumed is relatively small compared to the substrate uptake and does not account for full changes in metabolite spectrum. In the presence of [Co(*trans*-diammac)]^3+^, an average electron uptake of 2.51 mmol/L over 100 h was observed. At the same time about 9.6 mmol/L more lactate and 1.5 mmol/L more 2,3-BDO were produced compared to control fermentations. The stoichiometric balance of electron uptake does not account for the shift in metabolite production, however, it seems that electron consuming pathways such as lactate and 2,3-butanediol production are induced by the electron supply. Interestingly, this observation is only made in case of one mediator. While electron uptake was in the same range for all three molecules (2.77, 1.28 and 2.51 mmol/L for [Co(sep)]^3+^, [Co(AMMEsar)]^3+^ and [Co(*trans*-diammac)]^3+^, respectively) only [Co(*trans*-diammac)]^3+^ caused the discussed shift in carbon fluxes.

**Table 1:** Comparison of growth parameters, electron uptake, metabolite concentrations and yields of heterotrophic anaerobic fermentations of *C.* *autoethanogenum* in a bio-electrochemical system in the presence and the absence of three different Co-mediators

Condition	Control^a^	BES^b^	BES + [Co(sep)]^3+^	BES + [Co(AMMEsar)]^3+^	BES + [Co(*trans*-diammac)]^3+^
Growth rate (h^−1^)	0.053 ± 0.006	0.053 ± 0.006	0.037 ± 0.006	0.033 ± 0.006	0.06 ± 0.017
OD_600_ max	1.51 ± 0.18	1.11 ± 0.03	1.30 ± 0.12	1.22 ± 0.22	1.09 ± 0.14
Current max (mA)	–	0.07 ± 0.03	1.34 ± 0.24	1.07 ± 0.19	1.18 ± 0.20
*e* ^−^ uptake (mmol/L)	–	0.09 ± 0.01	2.77 ± 0.74	1.28 ± 0.16	2.51 ± 0.12
Final concentration (mmol/L)					
Acetate	46.43 ± 5.24	42.36 ± 0.58	48.22 ± 0.21	40.9 ± 7.38	21.11 ± 0.96
Lactate	0.28 ± 0.15	0.34 ± 0.04	0.34 ± 0.09	0.31 ± 0.11	9.87 ± 0.96
Ethanol	17.63 ± 2.95	21.57 ± 0.54	13.83 ± 1.33	19.74 ± 0.61	19.04 ± 2.4
2,3-BDO	0.73 ± 0.14	0.78 ± 0.05	0.48 ± 0.17	0.68 ± 0.19	2.21 ± 1.21
Yield (g/g_fructose_)					
BM	7.6 ± 1.2	6.86 ± 0.38	6.3 ± 0.36	7.74 ± 1.51	6.66 ± 1.64
Acetate	54.57 ± 4.31	50.51 ± 1.08	58.9 ± 0.36	52.06 ± 8.18	24.82 ± 2.35
Lactate	0.52 ± 0.27	0.60 ± 0.05	0.56 ± 0.21	0.56 ± 0.18	18.32 ± 2.27
Ethanol	12.76 ± 2.09	19.19 ± 0.23	10.89 ± 2.02	17.71 ± 2.28	15.69 ± 0.58
2,3-BDO	1.31 ± 0.28	0.89 ± 0.04	0.69 ± 0.07	1.09 ± 0.52	4.02 ± 1.99

Fermentation time = 100 h. All experiments were performed in biological triplicates

^a^Control fermentations were performed in the same reactor set up with electrodes at open circuit

^b^In BES condition the working electrode was poised at −0.603 V (vs SHE) w/o mediator

Observed rates of fructose and electron consumption and production rates of acetate, lactate, ethanol and 2,3-butanediol are summarized in Table 2. Noticeably, the rate of electrons that are taken up in the form of fructose is about 2–3 orders of magnitude higher compared to the cathodic electron uptake. Still, the same trend as for absolute metabolite concentrations is observed: Significant changes in metabolite production rates are observed exclusively for the low-potential mediator [Co(*trans*-diammac)]^3+^. Acetate production rate decreases from 5.68 mmol/g_CDW_ h in control fermentations to 4.05 mmol/g_CDW_ h, while production rates for lactate, ethanol and 2,3-butanediol increase from 0.02 to 1.98, from 2.49 to 3.45 and from 0.07 to 0.68 mmol/g_CDW_ h, respectively (refer to Table 2). Further, uptake rates for substrate as well as electrons are the highest in case of [Co(*trans*-diammac)]^3+^.

**Table 2:** Fructose and electron uptake rates and production rates of *C.* *autoethanogenum* observed under the different conditions

Condition	Uptake rate (mmol/g_CDW_ h)			Production rate (mmol/g_CDW_ h)			
	Fructose	Electrons from fructose^a^	Electrons from cathode	Acetate	Lactate	Ethanol	2,3-BDO
Control	−3.04 ± 0.29	−72.91 ± 6.96	–	5.68 ± 0.52	0.02 ± 0.01	2.49 ± 0.22	0.07 ± 0.01
BES	−2.96 ± 0.28	−71.02 ± 6.72	−0.01 ± 0.00	5.40 ± 0.07	0.02 ± 0.00	2.44 ± 0.06	0.05 ± 0.00
BES + [Co(sep)]^3+^	−2.96 ± 0.21	−70.98 ± 5.04	−0.19 ± 0.08	5.07 ± 0.02	0.02 ± 0.01	1.46 ± 0.14	0.04 ± 0.01
BES + [Co(AMMEsar)]^3+^	−3.36 ± 0.19	−80.56 ± 4.56	−0.07 ± 0.09	5.09 ± 0.32	0.03 ± 0.01	2.18 ± 0.07	0.13 ± 0.04
BES + [Co(*trans*-diammac)]^3+^	−5.25 ± 0.51	−125.92 ± 12.24	−0.23 ± 0.11	4.05 ± 0.18	1.98 ± 0.19	3.45 ± 0.33	0.68 ± 0.27

^a^24 mol electrons per mol fructose

While fructose presented the major carbon and electron source for *C.* *autoethanogenum* in the fermentations, the supplementary yeast extract present in the medium also contributed to metabolite production. As a result, conventional carbon balances as shown in Table 3 are overestimations (104–122%) when accounting for fructose consumption only. Therefore, a computational flux-optimization approach was used to perform a carbon and redox balance (detailed information given in methods section and Additional file 1: Table S1). While there are no suitable measurements to quantify the used yeast extract, it was possible to validate the dataset with two extreme scenarios. In the first analysis, the objective function was set to minimize the fructose uptake rate while obeying the measured rates for product formation and growth. The yeast extract was allowed to be used by the cells freely (compare "Methods" section for details). This analysis showed that in all cases the minimum required fructose uptake flux was smaller than the observed flux, meaning that depending on the uptake of yeast extract, there was surplus sugar available. At the same time, in this scenario, between 87.3 and 89.2% of the yeast extract would have been consumed over the analysis period. The carbon balance was closed by the optimization with about 11–16% of the balance made up of secreted CO_2_ or between 1.85 and 4.94 mmol/g_CDW_ h (see Table 3) and an energy surplus on top of growth-associated energy requirements of about 6.3–9.0 mmol_ATP_/g_CDW_ h was available for maintenance. This in silico analysis shows that the observed rates are feasible from a carbon, energy and redox balance points of view.

**Table 3:** Carbon balance accounting for metabolite production from fructose only (top) and Flux optimization of a *C. autoethanogenum* metabolic network using the experimental rates as constraints in CellNetAnalyzer (bottom)

Condition	Fructose consumed	Metabolites produced (mmol/L)					C-balance (%)
		Acetate	Lactate	Ethanol	2,3-BDO	CO_2_^a^	
Control	26.94	46.43	0.28	17.63	0.73	65.52	122.12
BES	27.24	42.36	0.34	21.57	0.78	65.49	120.83
BES + [Co(sep)]^3+^	27.24	48.22	0.34	13.83	0.48	63.01	116.28
BES + [Co(AMMEsar)]^3+^	25.47	40.90	0.31	19.74	0.68	62	122.32
BES + [Co(*trans*-diammac)]^3+^	25.99	21.11	9.87	19.04	2.21	44.57	104.71

Condition	Minimizing fructose consumption					Maximizing ATP maintenance			
	CO_2_ out	H_2_ out	ATP main	min fructose	YE (%)	CO_2_ out	H_2_ out	ATP main	YE (%)
Control	3.11	0.0	7.38	3.01	89.2	3.11	0.00	12.62	84.9
BES	3.19	0.0	7.19	2.67	87.3	3.01	0.00	12.09	53.6
BES + [Co(sep)]^3+^	1.85	0.0	6.29	2.23	88.0	3.16	3.10	7.81	0.0
BES + [Co(AMMEsar)]^3+^	2.68	0.0	7.38	2.74	88.0	3.83	2.76	9.96	0.0
BES + [Co(*trans*-diammac)]^3+^	4.94	0.0	9.02	4.51	88.6	5.02	0.81	12.75	0.0

Surplus carbon is allowed to leave the system as CO_2_, surplus redox power leaves as H_2_, ATP main(tenance) flux highlights energy surplus. Yeast extract (YE) flux is calculated on the assumed molecular composition and given as a percentage (%) of the total available yeast extract flux (compare M&M). Minimal fructose flux is the minimal rate of fructose uptake required to meet the observed production rates, based on optimal use of the YE. Apart from the YE all fluxes are given in (mmol/g_CDW_ h). The underlying network can be found in Additional file 1: Table S1

^a^CO_2_ production assumed from stoichiometric decarboxylation steps needed for production of acetate and ethanol (1 mol/mol) and 2,3-butanediol (2 mol/mol)

In the second flux optimization, ATP hydrolysis (also often referred to a maintenance reaction) was maximized. In this scenario, the fructose uptake flux was set to the observed value (Table 2). The control experiments still required yeast extract (84.9% for the control, 53.6% for the BES control, respectively), whereas in the case of the mediator experiments, fructose uptake was enough to satisfy all measured rates and maximize ATP production. This could point to a changed metabolism in the electrically enhanced cultures. Between 7.8 and 12.8 mmol_ATP_/g_CDW_ h could be synthesized on top of what was needed to make all the products including biomass; however, the model did not show a clear trend of higher ATP synthesis in the case of mediator experiments vs controls. The electrically enhanced experiments now showed a small surplus of electrons that was secreted as hydrogen (see Table 3), but the secreted amounts do not correlate with the observed electron-uptake fluxes (Table 2).

## Discussion

*Clostridium autoethanogenum* consumed electrons in the presence of all three mediators; however, the current profiles over time differ significantly (see Fig. 3). In all cases, a sharp current development around end-exponential growth phase was observed. The initial current development indicates that the bacteria were able to oxidize all three molecules, and thus successfully mediate electron transfer between the cathode and *C.* *autoethanogenum*. However, while the total maximum cathodic current was comparable for the different mediators (~−0.032 mA/cm^2^), the current profiles of [Co(sep)]^3+^ and [Co(AMMEsar)]^3+^ show that, in the presence of these mediators, this maximum current could not be maintained. Only in the presence of the low-potential mediator [Co(*trans*-diammac)]^3+^, the current profile remained constant at around the maximum value, indicating continuous consumption of electrons. A possible explanation for the differences in current profiles could be that in all cases, an initial reduction of cellular redox complexes could be obtained, thus leading to a negative current, but the electrons provided by [Co(sep)]^3+^ and [Co(AMMEsar)]^3+^ might not be low enough in potential to continuously drive thermodynamically favourable processes. Therefore, in both cases, the current quickly dropped again. Given the similarities in structure and properties of the mediators, the reported effect is believed to be truly potential dependent and impacts related to, e.g. lipophilic nature of the mediator can be excluded.

In terms of carbon shift, a recent study on *Clostridium pasteurianum* DSM 525 reported a comparable effect of extracellular electron supply during heterotrophic fermentations [24]. Electron supply via a cathode significantly shifted the metabolite spectrum towards alcohols and lactate while acetate production decreased. Similar to the observations for *C.* *autoethanogenum* made in this study, the current consumption is relatively small, and the major share of electrons recovered in the final end products originates from sugar oxidation. However, no investigation of required redox potentials to achieve this effect was undertaken. The authors assume that a small current consumption stimulates redox-balancing pathways such as lactate production. This could also explain the observations made for *C.* *autoethanogenum* in the presented study in case of [Co(*trans*-diammac)]^3+^.

**Fig. 5:**
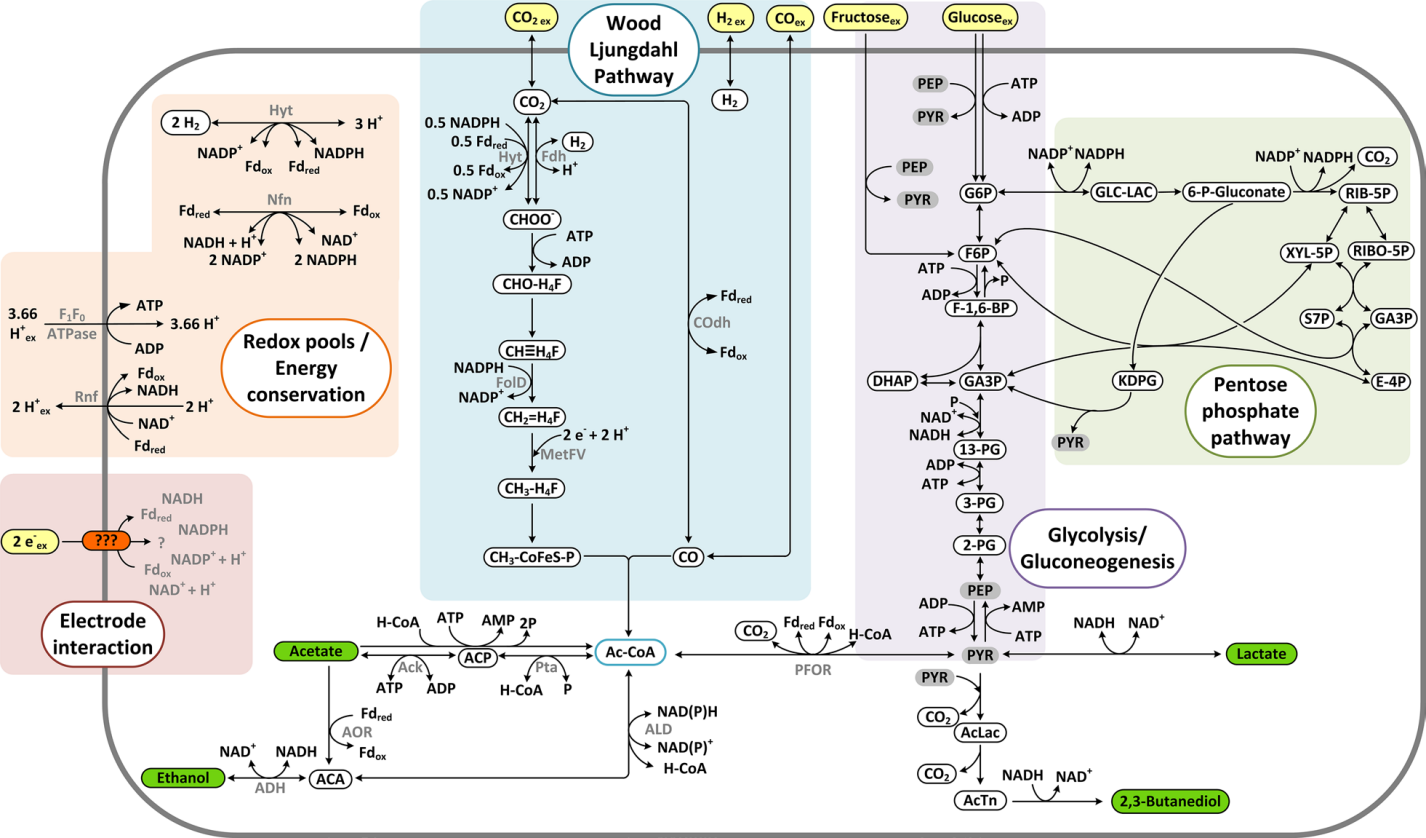
Network of the core carbon metabolism of *C.* *autoethanogenum* including redox and energy metabolisms.* Ack* Acetatekinase,* ADH* alcohol dehydrogenase,* ALD* CoA-dependent acetaldehyde dehydrogenase,* AOR* acetaldehyde:ferredoxin oxidoreductase,* COdh* carbon monoxide dehydrogenase,* Fdh* formate dehydrogenase,* FolD* methylene-H_4_F dehydrogenase,* Hyt* electron-bifurcating NADP- and ferredoxin-depended hydrogenase,* MetFV* Methylene-H_4_F reductase (co-factors remain unknown),* Nfn* electron-bifurcating ferredoxin-depended transhydrogenase,* PFOR* Pyruvate:ferredoxin oxidoreductase,* Pta* Phosphotransacetylase,* Rnf* membrane associated and energy conserving reduced ferredoxin:NAD^+^ oxidoreductase

Even though tools for metabolic and genetic engineering of acetogens are under intense development, there are major challenges when trying to shift the production away from the major product acetate. Not only do a large number of enzymes and regulatory mechanisms need to be engineered, but also pathway energetics have to be met [11]. If carbon flux is channelled away from ATP generating enzymes such as acetate kinase, a replacement energy source needs to be incorporated to provide a thermodynamically feasible scenario [25]. Here, we observe a decrease of acetate production by 55% by the simple approach of supplying a small amount of electricity in heterotrophic fermentations on fructose (~0.09 mol of electrons per mol of substrate); however, in terms of theoretically feasible ATP production, the flux optimization did not point to a clear advantage of electro-fermentation, highlighting that a regulatory switch triggered by the low-potential mediator might be at play. Increased fructose and electron uptake rates in case of [Co(trans-diammac)]^3+^ indicate an increase in metabolic activity. The simultaneous use of a heterotrophic carbon source and gases, called mixotrophy, was recently proposed as an option to increase yields for electron-dense products in acetogens [26]. In this concept, the Wood-Ljungdahl pathway is used to re-fix the two molecules of CO_2_ from decarboxylation reactions during glycolysis. The electrons needed for this fixation are supposed to be provided from reducing equivalents created during glycolysis as well. However, the reactions in WLP are NADPH and ferredoxin dependent, while glycolysis produces mainly NADH. If after glycolysis pyruvate is converted to Acetyl-CoA an additional reduced ferredoxin is obtained (refer to Fig. 5). The amount of available redox carriers for CO_2_ reduction could potentially be optimized by extracellular electron supply feeding into the ferredoxin pool. In the presented study, we observe a major metabolic shift away from acetate. However, the small electron uptake cannot stoichiometrically account for the shift in products, e.g. uptake of 2.5 mmol/L electrons in case of [Co(*trans*-diammac)]^3+^ result in an increase of 9.6 mmol/L lactate.

The characteristics of the redox metabolism of *C.* *autoethanogenum* are comparable to its close relative *C.* *ljungdahlii*. Figure 5 shows a simplified network of the core metabolism of *C.* *autoethanogenum* and its redox and energy metabolism. The heterotroph has no cytochromes and cellular redox pools are connected by specialized electron-bifurcating enzymes. A comprehensive, recent study by Mock et al. answered a range of open questions on bioenergetics of the organism by detailed determination of co-factors of metabolic redox reactions [25]. It was found that despite containing a variety of hydrogenases *C.* *autoethanogenum* seems to use only one active hydrogenase, which is electron bifurcating with ferredoxin and NADP^+^ as electron acceptors (Hyt: 2 H_2_ + Fd_ox_ + NADP^+^ ⇄ Fd_red_ + NADPH + 3 H^+^) [25]. Accordingly, most other redox reactions use NADPH or ferredoxin as co-factors (see Fig. 5). The second important electron-bifurcating enzyme is the Nfn complex that catalyses the reduction of NADP^+^ with reduced ferredoxin and NADH. Energy conservation other than substrate-level phosphorylation via acetate-kinase reaction (Ack) is provided via the Rnf-complex, a membrane-associated reduced ferredoxin:NAD^+^-oxidoreductase creating a proton gradient that drives ATP synthesis. The stoichiometric assumptions made for proton transport of Rnf complex and ATPase in Fig. 5 and the flux balance analysis are based on the analysis of Mock et al. [25]. It could be shown that Rnf activity contributes significantly to cellular energy yields, as deletion of the complex in *C.* *ljungdahlii* resulted in reduced growth on fructose and no growth on H_2_/CO_2_ [27].

We observed a metabolic shift in fructose fermentation towards lactate and 2,3-BDO in the presence of a redox mediator with sufficiently negative redox potential. Both products present NADH sinks (see Fig. 5) and therefore this might indicate that cathodic electron feed results in an increase of cellular NADH levels as seen for *C.* *pasteurianum* [24]. However, that assumption alone cannot explain the observed different effect between the three tested mediators. All three mediators should provide electrons at a sufficiently low potential to feed into the cellular NADH pool (around −280 mV required, see Fig. 2). Also, it is questionable why ethanol production, another NADH using pathway, is not affected. As discussed above, the redox-pools of *C.* *autoethanogenum* depend on ferredoxin, H_2_, NADH and NADPH. Figure 2b shows the comparison of the measured redox potentials of mediators and cellular redox compounds, and it can be seen that only a low-potential electron supply such as provided in the case of [Co(*trans*-diammac)]^3+^ theoretically could feed into the ferredoxin pool (−400 to −500 mV required) [28, 29]. The Rnf complex is ferredoxin dependent and the only known redox enzyme that is membrane associated in *C.* *autoethanogenum* [25]. In most cases of extracellular electron transport, membrane-bound proteins facilitate the electron transfer and because the mediators are charged molecules, a free diffusion into the cytoplasm is unlikely. Therefore, one possibility is an interaction of the redox mediators with the Rnf complex with only [Co(*trans*-diammac)]^3+^ providing a low enough potential to reduce ferredoxin. However, it remains unclear how exactly a little low-potential electron flow induces a major shift in carbon flows and why lactate might be a favorable overflow product, e.g. compared with ethanol. Further detailed studies are needed to elucidate the underlying mechanisms and provide evidence for extracellular electron transport routes in *C.* *autoethanogenum*.

## Conclusion

This study is the first to report electro-activity in the bio-chemical-producing *C.* *autoethanogenum*. Production in acetogenic organisms is usually challenged by cellular energy limitations if the target product is not acetate. These results demonstrate a significant shift of carbon fluxes away from acetate towards lactate and 2,3-butanediol by relatively small electron input.

The use of mediators with different redox potential revealed a positive correlation of the metabolic effect to the thermodynamic driving force. Only when electrons were provided at a potential low enough to reduce ferredoxin, a shift in metabolite spectra was observed. The analysis of electron-bifurcating enzymes in *C.* *autoethanogenum* identified the Rnf complex as membrane-bound and ferredoxin-dependent transhydrogenase as potential entry point for extracellular electrons into the metabolism. Given the low share of cathodic electrons within the overall electron uptake from substrates, however, it is possible that the effect is of regulatory origin. Further studies are needed to unveil the exact processes of electron transfer. Nevertheless, the presented work demonstrates a promising concept of using redox-mediating molecules to provide electrons as co-reducing power in heterotrophic fermentations to cause significant shifts towards reduced products at low electricity cost.

## Methods

### Culturing conditions

*Clostridium autoethanogenum* (DSM-10061) was obtained from the Leibniz Institute DSMZ-German Collection of Microorganisms and Cell Cultures, Braunschweig, Germany. The strain was cultured at 37 °C in modified DSMZ medium 879 inside a vinyl anaerobic chamber (Type A, Coy Laboratory Products, USA; atmosphere: 20% CO_2_, 4% H_2_, N_2_ to balance from Coregas, Australia). 1 L of modified medium 879 contained: 2-ethanesulfonic acid 20 g, NH_4_Cl 1 g, KCl 0.1 g, MgSO_4_ · 7 H_2_O 0.2 g, NaCl 0.8 g, KH_2_PO_4_ 0.1 g, CaCl_2_ · 2 H_2_O 0.02 g, yeast extract 2 g, trace element solution 141 10 mL, vitamin solution 141 10 mL, l-cysteine-HCl (100 mM) 10 mL, d-Fructose 5 g. From glycerol stocks first pre-cultures were inoculated inside the anaerobic chamber in 125 mL serum bottles with rubber stopper screw cap (Edwards, Australia) filled with either 50 or 100 mL media. First pre-cultures were sedimented by centrifugation (4000 rpm, 5 min, 37 °C, respectively, Centrifuge Sigma 3K30, DJB Labcare Ltd, Buckinghamshire, England) and re-suspended in fresh media (1:100 vol%). Cellular growth was monitored by measuring the optical density by UV–Vis Spectrophotometry (Genesys 10S, Thermo Fisher Scientific) and exponentially growing cells were harvested by centrifuging and washing in fresh media. This cell solution was used as inoculum for the main cultures in the BES. The entire process of centrifuging, washing and resuspending was performed under nitrogen atmosphere.

### Bio-electrochemical set up

The BES for the experimental characterization of MES was developed by optimizing a commercially available analytical cell kit from BioLogic (BioLogic, Science Instruments, France). The actual reactor vessel is a glass cell with working volume up to 150 mL, while during fermentations, a volume of 100 mL was used. The lid and fittings to connect probes and electrodes were custom made at a mechanical workshop at the University of Queensland from PEEK (Poly-ether ether ketone, purchased as raw material from E-Plas engineered & industrial plastics, Australia). Inert-gas atmosphere was achieved by constant flushing of the reactor headspace with nitrogen (flowrate 0.1 L/min), and the respective off-gas was led through a condenser chilled to 4 °C (custom made by LabGlass Pty Ltd, Brisbane, Australia) to minimize evaporation and stripping of volatile fermentation products. The working electrode was carbon cloth (CCP20 Fuel Cell Earth, Stoneham, USA) connected to a titanium wire (diameter 0.5 mm, purity 99.8%, Advent Research Materials, Oxford, England). The counter electrode was a titanium mesh connected to a titanium wire and was separated from the main reactor compartment by a cation-selective membrane (surface area: 2.55 cm^2^, CEM, Ultrex CMI7000, Membranes International Inc., USA). Titanium mesh and carbon cloth were cleaned by soaking three times in *iso*-propanol followed by rinsing with distilled sterile water before usage. The reference electrode was a commercial Ag/AgCl electrode in saturated KCl solution (+0.197 V vs SHE; BioLogic Science Instruments, France). Prior to every experiment, the complete BES was autoclaved (121 °C, 20 min, 2 atm) containing medium-omitting thermosensitive compounds, which were added later under sterile conditions.

### Cyclic voltammetry (CV) and chronoamperometry (CA)

Cyclic voltammetry measurements to characterize the redox mediators were performed in the BES described above using a multichannel potentiostat (Potentiostat/Galvanostat VSP or MPG2, BioLogic Science Instruments, France). The same electrode materials as used during electrically enhanced fermentations were used to record CVs. The anodic solution was 10× concentrated PBS buffer, while on the cathodic site, the different mediators were added at 1 mmol/L to the fermentation medium (modified medium 879, omitting carbon source). During the measurements, the potential of the working electrode was swept within the potential range of −1.0 to 0.0 V vs Ag/AgCl at a scan rate of 1 mV/s. The recorded scans were taken after five initial cycles to ensure reproducibility. Midpoint potentials were determined as the arithmetic average of anodic and cathodic peak potentials as determined from the analysis of the CV traces for each compound. Peak position was determined using the EC-LAB software (Version 10.40, BioLogic Science Instruments, France). During electrically enhanced fermentations, the BES were run in CA, whereby a constant potential of −0.603 V was applied on the carbon cloth working electrode. At this potential, no abiotic hydrogen production was observed, but full reduction of all tested mediators could be achieved (see Fig. 2 for CVs of medium blank and different mediators). The potential was applied prior to inoculation until a stable baseline current was achieved (refer to Fig. 3). Cumulative electric charge, that is, the amount of electrons consumed (Coulombs), was calculated for each fermentation by integration of the current profile versus time logged by the potentiostat after subtraction of the corresponding baseline current. Electric charge (*Q*) was converted into moles of electrons (*n*(*e* ^−^)) applying Faraday’s law with *F* being Avogadro’s number (96,484 C/mol): $Q = n\left( {e^{ - } } \right) * F$.

### High-pressure liquid chromatography

To measure metabolite profiles fermentation samples were analysed via HPLC. Organic acids were detected from pure cell-free samples, sugar from 1/10 dilutions. The used device (Agilent 1200 Series, Agilent Technologies, Santa Clara, USA) was a reverse-phased chromatography and consisted of seven different modules (G1312A Binary pump, G1379B degasser, G1367B high performance autosampler, G1330B FC/ALS (thermostat), G1365B column compartment (thermostat), G1365B multiple wavelength detector (MWD) and G1362A refractive index detector). The column Phenomenex Rezex RHM Monosaccharide H^+^ had the dimension of 300 × 7.8 mm plus an up-streamed guard column. The HPLC was driven with a mobile phase of 100% 0.008 N sulphuric acid at a flow rate of 0.6 mL/min. The maximum pressure was set to 80 bar and the column temperature to 70 °C. 30 µL of every sample was injected and had a run time of 25 min. The wavelength of the MWD was 210 nm.

### Flux optimization

In order to gain a better understanding of the impact of electron supply to the metabolic network, a flux optimization was performed with CellNetAnalyzer [30, 31]. The version 2015.1 was obtained under academic-free licence from http://www2.mpi-magdeburg.mpg.de/projects/cna/cna.html. The metabolic network containing the core metabolism comprised 92 metabolic reactions and 89 metabolites. Reversible reactions and parallel pathways were constrained when futile loops were observed. The network reaction stoichiometry is provided in Additional file 1: Table S1. Yeast extract was present in the medium. While yeast extract can have a diverse composition, assuming a composition and then estimating the impact on the optimization still allows to explore the boundaries of the system and to get an estimate on the feasibility of the measured rates. We assumed that the yeast extract would consist of around 58% protein (proteins, peptides, amino acids), and 14.2% RNA about 2.5% carbohydrates and the balance ashes, using the known precursor demand to synthesize the macromolecules in baker’s yeast [32], we estimate total available precursors (assuming the cells can use all components). The analysis is to be seen as a tool to estimate the overall feasibility of the analysed dataset. The optimization was set first to minimize fructose consumption and second to maximize ATP maintenance energy.

## Additional file

**Additional file 1: Table S1.** The metabolic model of* C. autoethanogenum* that was used for flux balance analysis.

## Abbreviations

Ack: acetatekinase
ADH: alcohol dehydrogenase
ALD: CoA-dependent acetaldehyde dehydrogenase
AOR: acetaldehyde:ferredoxin oxidoreductase
BES: bio-electrochemical system
COdh: carbon monoxide dehydrogenase
CV: cyclic voltammetry
Fdh: formate dehydrogenase
FolD: methylene-H_4_F dehydrogenase
Hyt: electron-bifurcating NADP- and ferredoxin-dependent hydrogenase
MES: microbial electrosynthesis
MetFV: methylene-H_4_F reductase (co-factors remain unknown)
Nfn: electron-bifurcating ferredoxin-depended transhydrogenase
OD_max_: maximum optical density
PFOR: pyruvate:ferredoxin oxidoreductase
Pta: phosphotransacetylase
Rnf: membrane associated and energy conserving reduced ferredoxin:NAD^+^ oxidoreductase
SHE: standard hydrogen electrode
WLP: Wood-Ljungdahl pathway
